# Toward Universal Photodynamic Coatings for Infection Control

**DOI:** 10.3389/fmed.2021.657837

**Published:** 2021-07-28

**Authors:** C. Roland Ghareeb, Bharadwaja S. T. Peddinti, Samantha C. Kisthardt, Frank Scholle, Richard J. Spontak, Reza A. Ghiladi

**Affiliations:** ^1^Department of Chemistry, North Carolina State University, Raleigh, NC, United States; ^2^Department of Chemical and Biomolecular Engineering, North Carolina State University, Raleigh, NC, United States; ^3^Department of Biological Sciences, North Carolina State University, Raleigh, NC, United States; ^4^Center for Advanced Virus Experimentation, North Carolina State University, Raleigh, NC, United States; ^5^Department of Materials Science and Engineering, North Carolina State University, Raleigh, NC, United States

**Keywords:** antimicrobial, Coronavirus, coatings, photodynamic inactivation, photosensitizer, polymer, singlet oxygen, *Staphylococcus aureus*

## Abstract

The dual threats posed by the COVID-19 pandemic and hospital-acquired infections (HAIs) have emphasized the urgent need for self-disinfecting materials for infection control. Despite their highly potent antimicrobial activity, the adoption of photoactive materials to reduce infection transmission in hospitals and related healthcare facilities has been severely hampered by the lack of scalable and cost-effective manufacturing, in which case high-volume production methods for fabricating aPDI-based materials are needed. To address this issue here, we examined the antimicrobial efficacy of a simple bicomponent spray coating composed of the commercially-available UV-photocrosslinkable polymer *N*-methyl-4(4'-formyl-styryl)pyridinium methosulfate acetal poly(vinyl alcohol) (SbQ-PVA) and one of three aPDI photosensitizers (PSs): zinc-tetra(4-*N*-methylpyridyl)porphine (ZnTMPyP^4+^), methylene blue (MB), and Rose Bengal (RB). We applied these photodynamic coatings, collectively termed SbQ-PVA/PS, to a variety of commercially available materials. Scanning electron microscopy (SEM) and time-of-flight secondary ion mass spectrometry (ToF-SIMS) confirmed the successful application of the coatings, while inductively coupled plasma-optical emission spectroscopy (ICP-OES) revealed a photosensitizer loading of 0.09-0.78 nmol PS/mg material. The antimicrobial efficacy of the coated materials was evaluated against methicillin-susceptible *Staphylococcus aureus* ATCC-29213 and human coronavirus strain HCoV-229E. Upon illumination with visible light (60 min, 400-700 nm, 65 ± 5 mW/cm^2^), the coated materials inactivated *S. aureus* by 97-99.999% and HCoV-229E by 92-99.999%, depending on the material and PS employed. Photobleaching studies employing HCoV-229E demonstrated detection limit inactivation (99.999%) even after exposure for 4 weeks to indoor ambient room lighting. Taken together, these results demonstrate the potential for photodynamic SbQ-PVA/PS coatings to be universally applied to a wide range of materials for effectively reducing pathogen transmission.

## Introduction

As one of the most catastrophic health crises in modern history, the global COVID-19 pandemic caused by the SARS-CoV-2 virus has pummeled strong national economies, imposed unprecedented social restrictions and, above all else, claimed over 3.8 million lives worldwide (with over 600,000 now dead in the U.S. alone) at the time of this writing (1). The virus is primarily transmitted *via* aerosolized droplets that disperse into the air during speaking, coughing, or sneezing, thereby necessitating the use of facemasks and other protective personal equipment (PPE). Recent studies by Munster et al. (2) have demonstrated, however, that the virus remains stable on several surfaces for long periods of time (up to 2-3 days on stainless steel and unspecified plastic), whereas Chin et al. (3) have measured even longer stability times, including 7 days on surgical masks. These results indicate that SARS-CoV-2 may also spread by direct contact with contaminated surfaces, including PPE, which is particularly worrisome considering how readily the pathogen spreads within hospitals and related healthcare settings. If the COVID-19 pandemic represents an acute healthcare challenge due to uncontrollable pathogen spread, then the chronic problem invariably translates to nosocomial/hospital acquired infections (HAIs): according to the CDC (4), 1 out of every 20 hospital patients is affected by nosocomial infections, resulting in 100,000 deaths annually in the U.S. alone. The reasons behind these numbers are well-established: improperly disinfected surfaces retain pathogens that infect new hosts, properly disinfected surfaces are quickly and easily re-contaminated by either healthcare personnel or the patients themselves, and pathogens are able to reside on non-routinely cleaned surfaces (e.g., hospital curtains and linens) and remain infectious for long periods of time (5), estimated to be on the order of weeks in some cases. For instance, nosocomial pathogens, including *A. baumannii* and *Staphylococcus aureus*, have been reported (6, 7) to survive from weeks to months on inanimate surfaces. Medical textiles, in particular, highlight the need for inherently antimicrobial materials to prevent pathogen transmission, either directly or indirectly, between the hospital environment, patients and healthcare workers (8–10).

Collectively, HAIs and the COVID-19 pandemic are driving a feverish search for long-life antimicrobial materials for efficient infection control. While a variety of approaches are being explored for this purpose (11–22), one such method to produce self-disinfecting materials and provide an opportunity to inactivate various microbes on surfaces prior to human infection for prolonged periods of time (but which itself is unlikely to lead to the development of drug-resistance) is *a*ntimicrobial *p*hoto*d*ynamic *i*nactivation (aPDI), a branch of photomedicine that employs light, air, and a photosensitizer (PS) to generate radicals (Type I) and/or reactive oxygen species (ROS, primarily biocidal ‘singlet' oxygen; Type II) as the microbiocidal agent(s) (23–29). Using this approach, we have recently formulated a photodynamic coating that can be applied to a wide range of substrates (30). Comprised of the UV-photocrosslinkable polymer *N*-methyl-4(4'-formyl-styryl)pyridinium methosulfate acetal poly(vinyl alcohol) (SbQ-PVA) and the well-studied PS zinc-tetra(4-*N*-methylpyridyl)porphine (ZnTMPyP^4+^) (31–33), both of which are commercially available, the photodynamic coating was easily applied to nylon-6 microfibers through both spray and dip coating. Antimicrobial studies confirmed photodynamic inactivation against methicillin-susceptible *S. aureus* (MSSA) and antibiotic-resistant *E. coli* (AREC) with population reductions of 99.9999+% and 99.6%, respectively, after exposure to visible light (400-700 nm; 65 mW/cm^2^) for 60 min. Critically, the spray-coated fibers were capable of inactivating human coronavirus strain 229E (a BSL-2 surrogate for SARS-CoV-2) with visible light (400–700 nm, 80 mW/cm^2^) illumination by 99.9+% (three log units, the minimum detection limit in that study).

Although the above results from the initial study are promising, several outstanding questions remain regarding the applicability and utility of this approach. Namely, can the SbQ-PVA/PS photodynamic coating be applied to a variety of commercially available materials (e.g., fabrics and linens), can other commercially available photosensitizers be employed in addition to ZnTMPyP^4+^, and what is the longevity of these photodynamic materials when exposed to ambient room light for prolonged periods of time (e.g., weeks)? To address these and other questions, we present here results expanding the application of this facile coating procedure to a wider variety of commercially available materials. We further investigated the efficacy of the SbQ-PVA/PS coating with both methylene blue (MB) and Rose Bengal (RB) PSs, and evaluated the efficacy of the photodynamic coatings for up to 1 month of exposure to ambient room light for antiviral activity against human coronavirus strain 229E. The results presented herein establish aPDI spray coatings such as SbQ-PVA/PS as an economically viable and broadly applicable means for imparting self-disinfecting properties to a wide variety of common fibrous materials.

## Materials and Methods

### Materials

The ZnTMPyP^4+^ tetrachloride PS was purchased from Frontier Scientific, while MB and RB were obtained from Acros Organics. The photocrosslinkable SbQ-PVA polymer with 4.1 mol% functional SbQ groups was supplied by Polysciences, Inc. The following materials were donated by Vescom America: Capri (polyester curtain fabric), Deans (polyester upholstery), Husk (proprietary wallcovering), PRU-86364 (proprietary wallcovering), and Wolin (a 75/20/5 w/w/w/ wool/polyester/polyamide upholstery combination). Buffer salts for the preparation of phosphate-buffered saline (PBS) solution and ultrapure nitric acid for ICP-OES analysis were purchased from Fisher Scientific. Tryptic soy broth was purchased from Teknova, and all media and buffer solutions were prepared in ultrapure water provided by an Easypure II system (Barnstead).

### Coating Protocol

The coating protocol was performed as previously described (30) with minor modifications. The SbQ-PVA was dissolved in deionized (DI) water at a fixed concentration of 10% w/v SbQ-PVA/water and stirred until fully dissolved according to the unaided eye. Subsequently, photosensitizers (ZnTMPyP^4+^, MB, or RB) were dissolved into the aqueous SbQ-PVA solution to achieve a constant PS loading of 1 wt% in PS/SbQ-PVA, and the resulting solution was stirred for an additional 15 min prior to coating. The Vescom materials were cut into squares measuring 8 x 8 cm, with one side of each spray-coated until saturated (between 1 and 4 ml of solution) using a Master Airbrush Model G22 with a 0.3 mm fluid tip. The samples were then cured in the presence of a MelodySusie UV light (36 W, 365 nm) for 60 min, and the coating process was repeated on the opposite coupon side. A secondary “seal” coat consisting of ~0.05 ml of a PS-free aqueous SbQ-PVA solution was then applied on each side, followed by UV-curing for an additional 30 min. Following UV-curing, the coated samples were cut with a hole punch into circles measuring either ~0.5 or 1 cm in diameter for antiviral and antibacterial assays, respectively. The sample discs were then dip-coated in a 10% w/v PS-free aqueous SbQ-PVA solution to apply a capping layer, and were further UV-cured until dry. For reference, PS-free control samples were prepared solely from dip-coated Vescom discs. After the final UV cure, all the discs were washed in DI water overnight and thoroughly dried prior to characterization and conducting the aPDI assays.

### Characterization

#### Colorimetric Analysis

Colorimetric analysis was performed at the NC State University Color Science and Imaging Laboratory. To prevent the light from shining through the fabrics, each sample was folded into layers before performing the colorimetric analysis on a Datacolor 650 spectrophotometer equipped with a D65 light source and an aperture of 9 mm at a viewing angle of 10°.

#### Time-of-Flight Secondary Ion Mass Spectrometry

Time-of-flight secondary ion mass spectrometry (ToF-SIMS) analysis was conducted on coated and uncoated Vescom materials using an IONTOF ToF-SIMS V instrument equipped with a 25 kV bismuth ion (Bi^+^) sputtering gun. High-resolution ion-specific images were collected wherein the C_2_H_3_${\text{O}}_{2}^{-}$ ion served to indicate the presence of the SbQ-PVA polymer coating.

#### Scanning Electron Microscopy

The surface morphologies of the coated Vescom samples were examined by scanning electron microscopy (SEM) performed on either a variable-pressure Hitachi S3200N microscope equipped with an Oxford energy-dispersive X-ray spectroscopy (EDS) detector (for Wolin, Deans, Capri, PRU, and Husk), or an FEI Verios 460L FESEM microscope (for Wolin at 50x magnification). The sample discs were mounted on aluminum stubs with carbon tape and sputtered with ~35 nm of Au/Pd to reduce charging. Images were acquired at an accelerating voltage of 20 kV and, in the case of specimens imaged on the Hitachi microscope, a column pressure of 30 Pa N_2_.

#### Trace Metal Analysis

Trace metal analysis was performed by the NC State University Environmental and Agricultural Testing Service. The total loading of ZnTMPyP^4+^ on each coated Vescom sample was determined by inductively coupled plasma-optical emission spectroscopy (ICP-OES). Following a previously published protocol (34), circular samples measuring ~1 cm in diameter were weighed and dissolved in 10 ml ultrapure nitric acid, stirred overnight, centrifuged (at 4,121*g*), and filtered prior to Zn analysis on a Perkin Elmer 8000 ICP-OES.

### Antimicrobial Photodynamic Inactivation Studies

#### Bacterial Inactivation

Antibacterial photodynamic inactivation assays were performed with Gram-positive methicillin-susceptible *S. aureus* 29213 (MSSA). Cultures were grown in 5 ml tryptic soy broth (TSB) incubated at 37°C in an orbital shaker operated at 250 rpm, and its optical density (OD) was monitored at 600 nm with a Genesys 10 UV scanning spectrophotometer. Cultures were grown to the mid-log phase with an OD of ~0.4, which corresponds to a bacterial concentration of 1-4 x 10^8^ colony-forming units per mL (CFU/ml). Afterwards, the culture was centrifuged for 5 min (at 3,374*g*) and the supernatant was subsequently discarded. The resultant bacterial pellets were re-suspended in 5 ml PBS prior to the aPDI assay. Vescom sample discs (~1 cm diameter) were fitted into the well-bottoms of a 24-well plate (3 PS-containing samples, 1 PS-free control sample), to which 200 μl of the resuspended bacterial suspension was added and uniformly deposited on top of each sample. An identical plate protected from light with aluminum foil was prepared for the purpose of a dark control. Illumination was provided by a LumaCare LC-122 incoherent visible light source equipped with an OSRAM 64653 HLX Xenophot bulb (250 W, 24 V) and employing a LUM V fiber optic probe (400–700 nm band pass filter) with 95 ± 3% average transmittance. The fluence rate of the light source was measured with an Orphir Optronics Ltd. Orion power meter. A target illumination intensity of 65 ± 5 mW/cm^2^ was applied in accordance with our previous studies (30, 35–44). After addition of the bacterial suspension to each well, the well-plate was illuminated for 60 min, after which 40 μl was withdrawn from each of the wells and added to a 360 μl PBS aliquot to serve as a 1:10 dilution. This procedure was repeated five times to generate six ten-fold serial dilutions for each well. A 10 μl aliquot from each dilution was pipetted onto six-column-gridded square plates that were previously prepared with antibiotic-free TSB/agar, and then incubated overnight at 37°C. Colony-forming units were counted and the corresponding level of bacterial inactivation was calculated by dividing the CFU/ml count of the illuminated samples by either the corresponding dark or PS-free controls. All studies were performed in triplicate, and statistical significance (*p*) was assessed using an unpaired student's two-tailed *t*-test (in which statistical significance is established when *p* < 0.05). The minimum detection limit (MDL) for these antibacterial studies was 0.0001% survival.

#### Viral Inactivation

The human coronavirus HCoV-229E was grown to a titer of 10^9^ TCID_50_/ml on the human hepatocarcinoma (Huh-7) cell line in cell growth media (DMEM, 1% antibiotics, 10% fetal bovine serum, FBS) at 35°C. The spray-coated Vescom samples (6 mm diameter) were fitted into the well-bottoms of a 96-well plate, and 25 μl of virus suspension (free of the host Huh-7 cells) was added to the wells. A set of dark controls wrapped in aluminum foil was left unexposed to light for 60 min. For samples exposed to light, the illumination intensity was 65 ± 5 mW/cm^2^. After illumination for 60 min, 75 μl of infection media (DMEM 1% antibiotics, 1% FBS, 1% HEPES buffer) were added, and the virus was eluted by triturating several times, followed by rapid transfer to new wells. Virus suspensions were immediately diluted serially ten-fold, and 50 μl of four replicates of each dilution were used to infect Huh-7 cells seeded the previous day at a density of 10^4^ cells per well in a TCID_50_ assay protocol. The plates were incubated at 35°C with 5% CO_2_. After 96 h, the cytopathic effect was monitored by visual inspection, and resulting log_10_TCID_50_/ml values (MDL of 2.8 log_10_ TCID_50_/ml) were calculated according to the Spearman-Kaerber method. All studies were performed in triplicate. Studies were performed in a similar manner for human adenovirus-5 and feline calicivirus. Human adenovirus-5 (hAd-5) was grown on A549 human lung carcinoma cells in DMEM supplemented with 10% FBS and antibiotics. Titration was performed by TCID50 assay on the same cell line. CPE was assessed visually at day 5 post infection. Feline calicivirus (FCV) was propagated on Crandall-Reese Feline Kidney cells (CRFK) in DMEM supplemented with 10% FBS and antibiotics. TCID50 assays were performed for titration on CRFK cells and CPE scored visually at day 3 post infection.

#### Longevity Studies

For each photosensitizer (MB, RB, and ZnTMPyP^4+^), a total of 15 samples (6 mm diameter) of Husk material coated with SbQ-PVA/PS were prepared. Of these 15 samples, three were stored in the dark at ambient temperature for later testing as non-photobleached materials (at t = 0). The 12 remaining samples were placed in a petri dish on a tabletop and continuously exposed to ambient laboratory room light (fluorescent) weekly for 4 weeks. During this time, three samples were collected and stored in the dark at ambient temperature until evaluation. At the conclusion of 4 weeks, the photo-aged and non-photobleached (t = 0) samples, as well as dark controls and PS-free controls, were evaluated three times: first, as described above in section Viral Inactivation; second, taking these one-time evaluated samples, freezing them at −20°C for 18 days, thawing each sample followed by washing in 200 μl PBS, and then repeating the viral inactivation assay; and third, after washing, repeating the antiviral assay on the twice-evaluated samples. These three-time evaluated samples were compared to freshly prepared materials.

## Results

### Materials Coating

In our previous study (30), SbQ-PVA/ZnTMPyP^4+^ was successfully applied by both spray- and dip-coating methods to relatively uniform nylon-PA6 spunbond fibers. To determine if SbQ-PVA/PS coatings could be applied to a broader range of materials commonly utilized in areas such as hospital waiting rooms and patient rooms, a variety of commercially-available materials (from Vescom America) having applications from curtains to upholsteries and wallpaper were examined. Here, we primarily employed ZnTMPyP^4+^ for characterization and antimicrobial evaluation for comparative purposes to our previous studies employing this and related tetrapyrrole photosensitizers (30, 33, 34, 37–40, 43, 44). Other photosensitizers, e.g., MB and RB, were also evaluated for antimicrobial efficacy and will be discussed further below.

**Figure 1:**
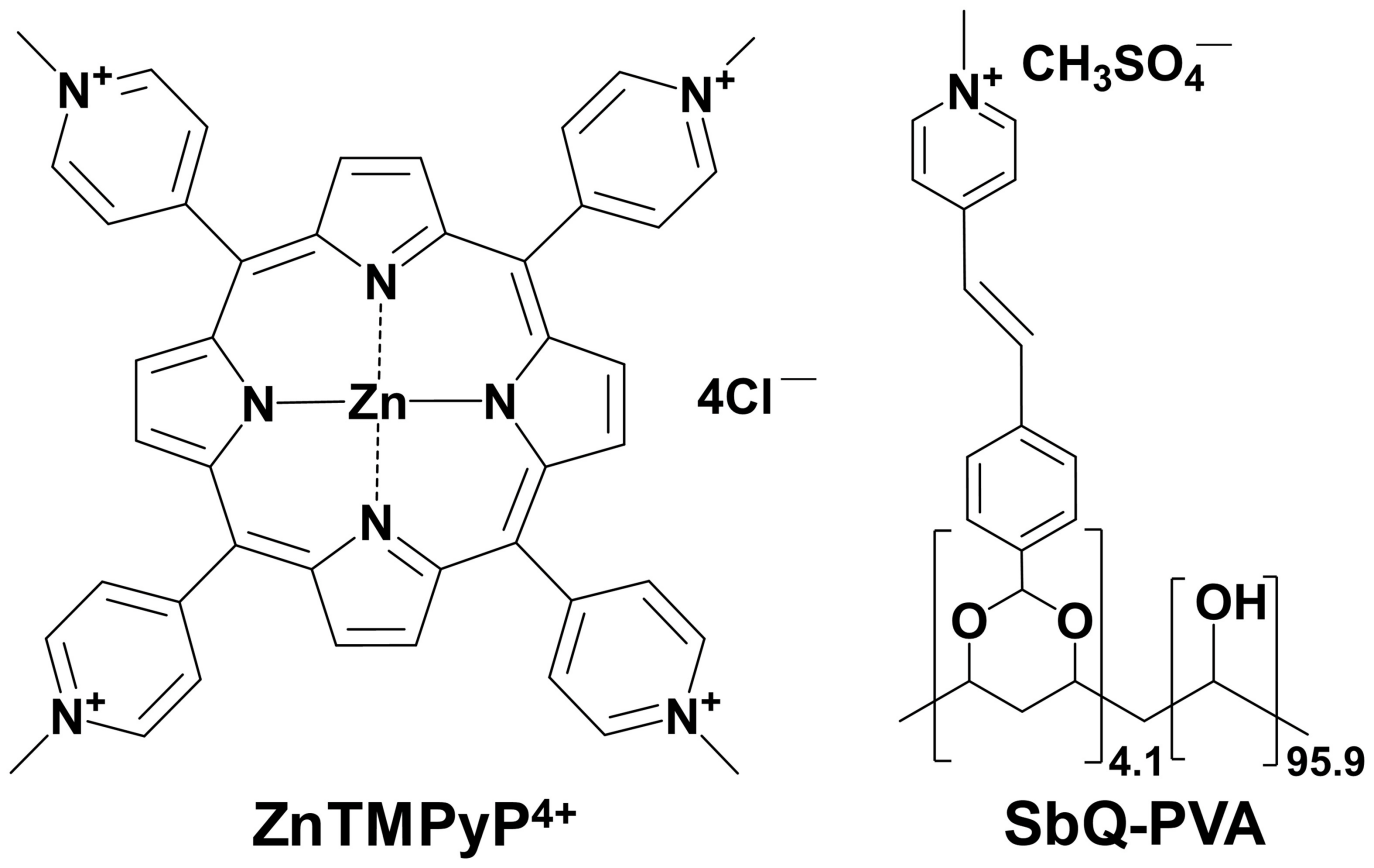
Chemical structures of (left) the ZnTMPyP^4+^ photosensitizer and (right) the water-soluble, photocrosslinkable SbQ-PVA polymer.

Each Vescom material was prepared using the same method in which the bicomponent SbQ-PVA/PS (Figure 1) mixture was applied to both sides of an 8 x 8 cm sample *via* spray coating, followed by UV curing. Two separate “sealant” coats were then applied utilizing SbQ-PVA alone: the first by spray coating to ensure the photosensitizer was fully embedded within the polymer matrix to prevent PS leaching, and the second by dip coating after samples were cut to size to further seal the fibers from possible damage incurred during specimen trimming. After preparation, the materials were washed overnight to remove any unbound PS, although this was found to be unnecessary (i.e., no significant PS leaching was observed spectroscopically).

**Figure 2:**
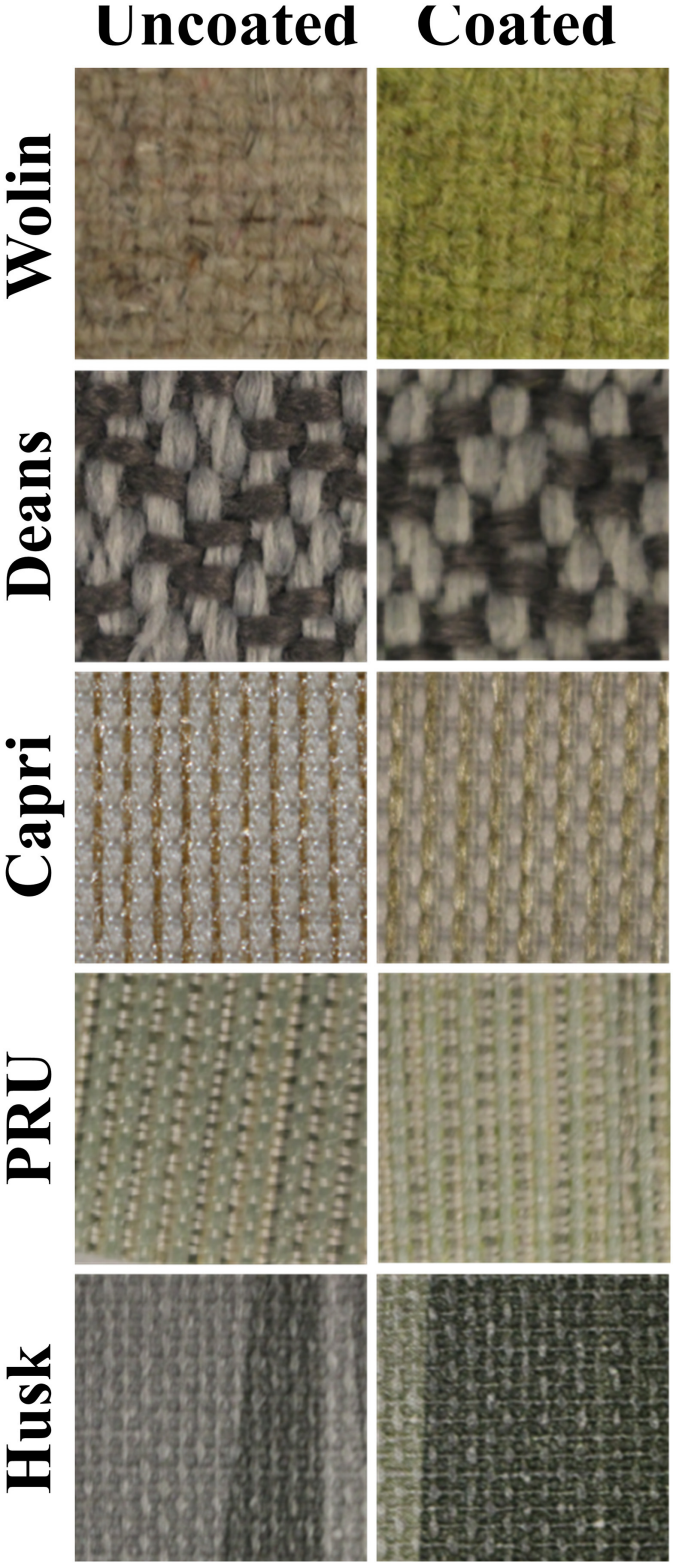
Photographic images of Vescom materials without (left) and with (right) the SbQ-PVA/ZnTMPyP^4+^ coating.

**Figure 3:**
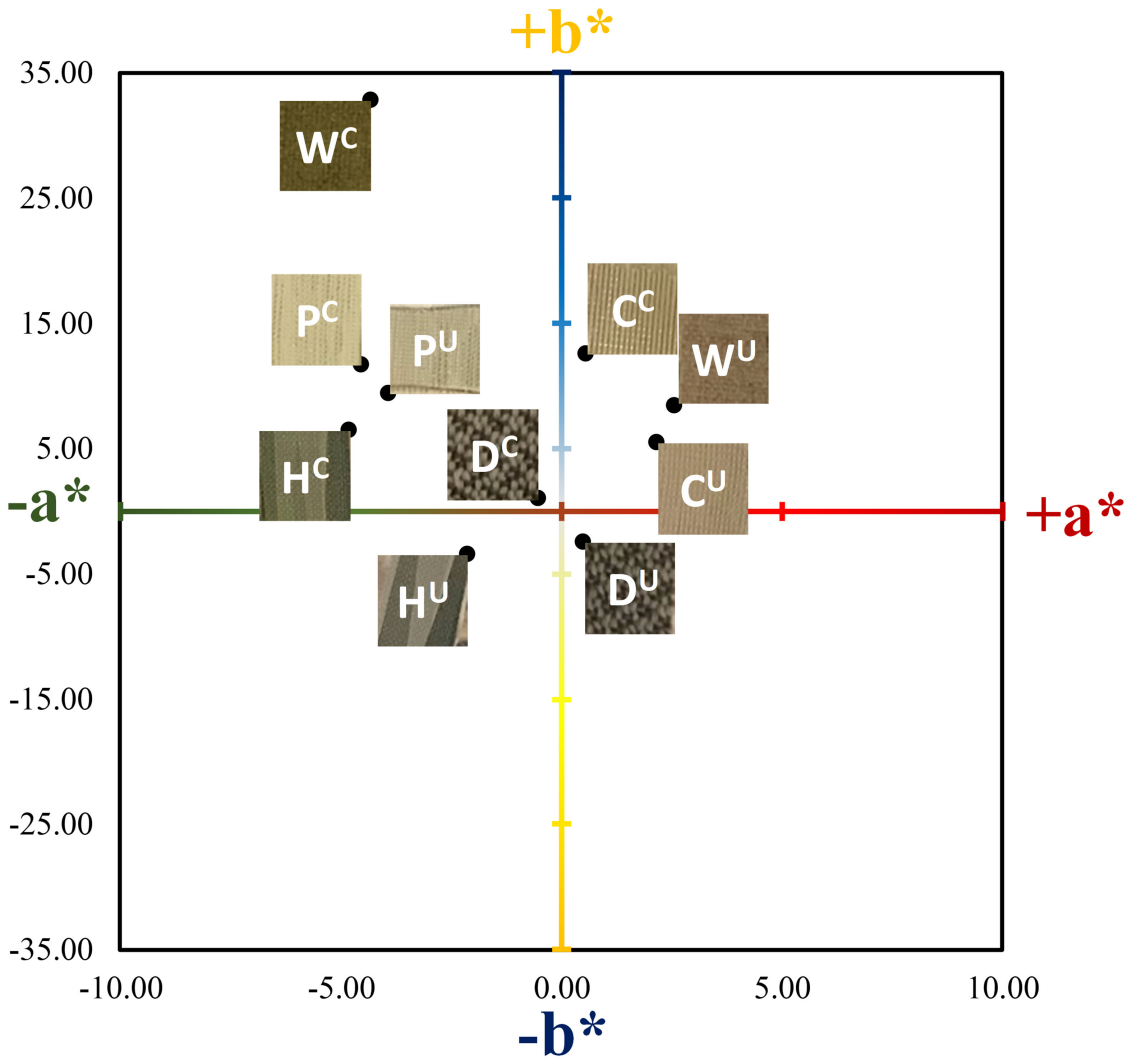
Corresponding coordinate positions of the Vescom materials in *CIELab* color space for a* and b*. The L* values (white/black) were not plotted for simplicity. The superscripts U and C denote uncoated and coated (SbQ-PVA/ZnTMPyP^4+^) materials, respectively. Labels are as follows: C, Capri; D, Deans; H, Husk; P, PRU-86364; W, Wolin.

As can be seen in Figure 2, the presence of the ZnTMPyP^4+^ photosensitizer can be visually observed in the coated samples from their increased green hue when compared to the uncoated samples. Beyond this change in color, the materials remained unchanged with regard to human touch/feel. Colorimetric analysis (*CIELab* values) was performed on both coated and uncoated samples (Table 1) to quantitatively assess the color change upon PS addition. As expected, due to the absorption properties of the ZnTMPyP^4+^ PS (λ_max_ = 436 nm), there is an overall shift toward green (toward –a^*^, +b^*^; Figure 3) in all samples post-coating, consistent with the presence of the PS. In line with the visual changes depicted in Figure 2, the Wolin specimen exhibited the greatest changes in *CIELab* values after coating, followed by the Husk and Capri materials that both showed similar overall green shifts, while the Deans and PRU samples displayed the smallest overall shift. While this analysis confirms the overall presence of the photosensitizer qualitatively, it is not indicative of the quantitative PS loading (*vide infra*), as the initial color and hue of each material likely impacts the *CIELab* values.

**Table 1:** CIE*Lab* Colorimetric Analysis of Uncoated and SbQ-PVA/ZnTMPyP^4+^ Coated Vescom Materials.

**Specimen**	CIE L*		CIE a*			CIE b*			**K/S**	
	**Uncoated**	**Coated**	**Uncoated**	**Coated**	Δa*	**Uncoated**	**Coated**	Δb*	**Uncoated**	**Coated**
Wolin	65.78	55.13	2.58	−4.30	−6.88	8.41	32.76	24.35	1.22	3.48
Deans	54.40	51.24	0.52	−0.51	−1.03	−2.46	1.01	3.47	1.34	1.92
Capri	67.65	69.84	2.17	0.59	−1.58	5.43	12.50	7.07	0.75	0.91
PRU	73.63	73.52	−3.90	−4.51	−0.61	9.34	11.67	2.33	0.61	0.68
Husk	46.91	59.01	−2.10	−4.78	−2.68	−3.45	6.47	9.92	2.69	1.60

### Characterization

#### Polymer Coating

**Figure 4:**
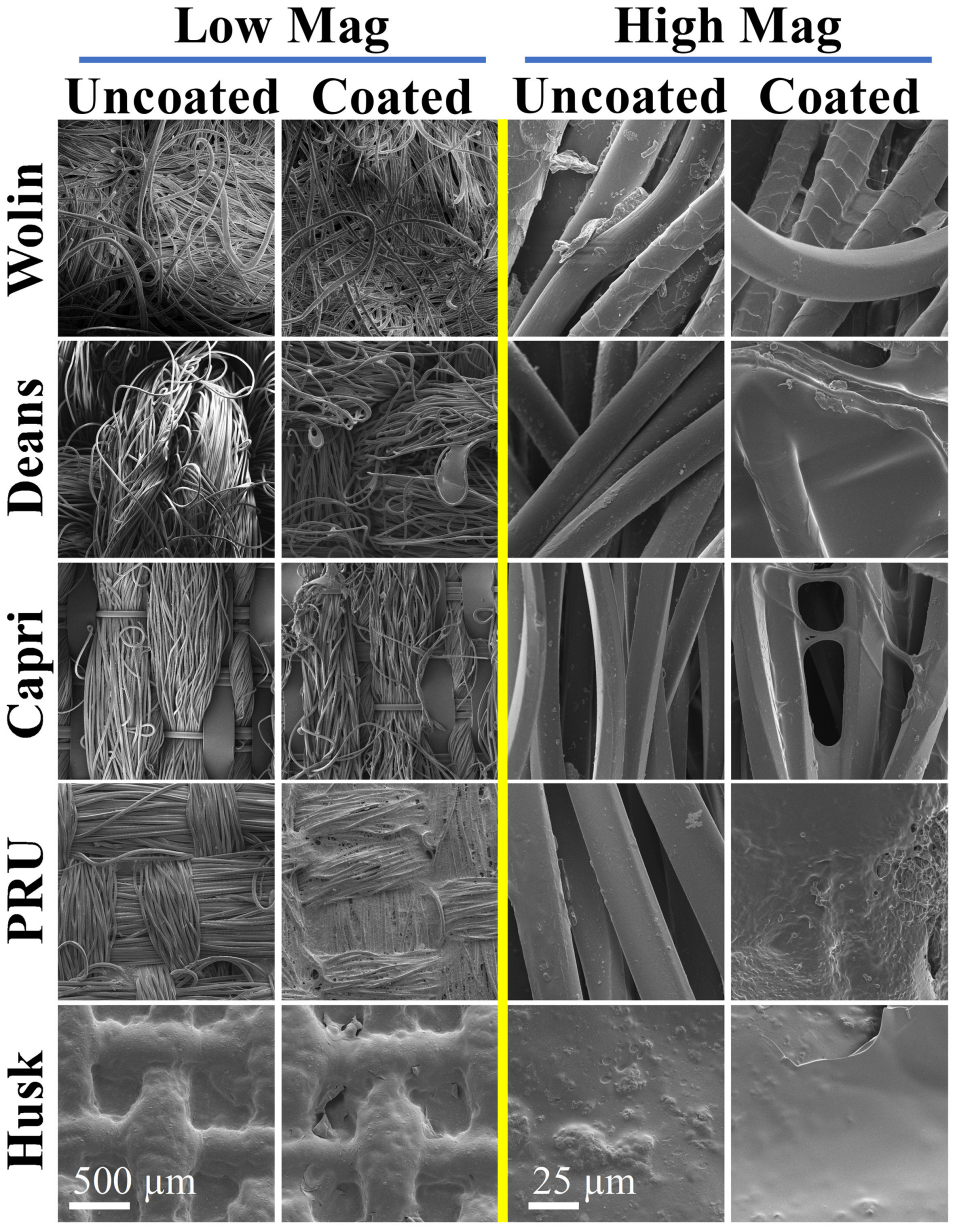
SEM images of uncoated and coated (SbQ-PVA/ZnTMPyP^4+^) Vescom materials at low (left) and high (right) magnifications.

**Figure 5:**
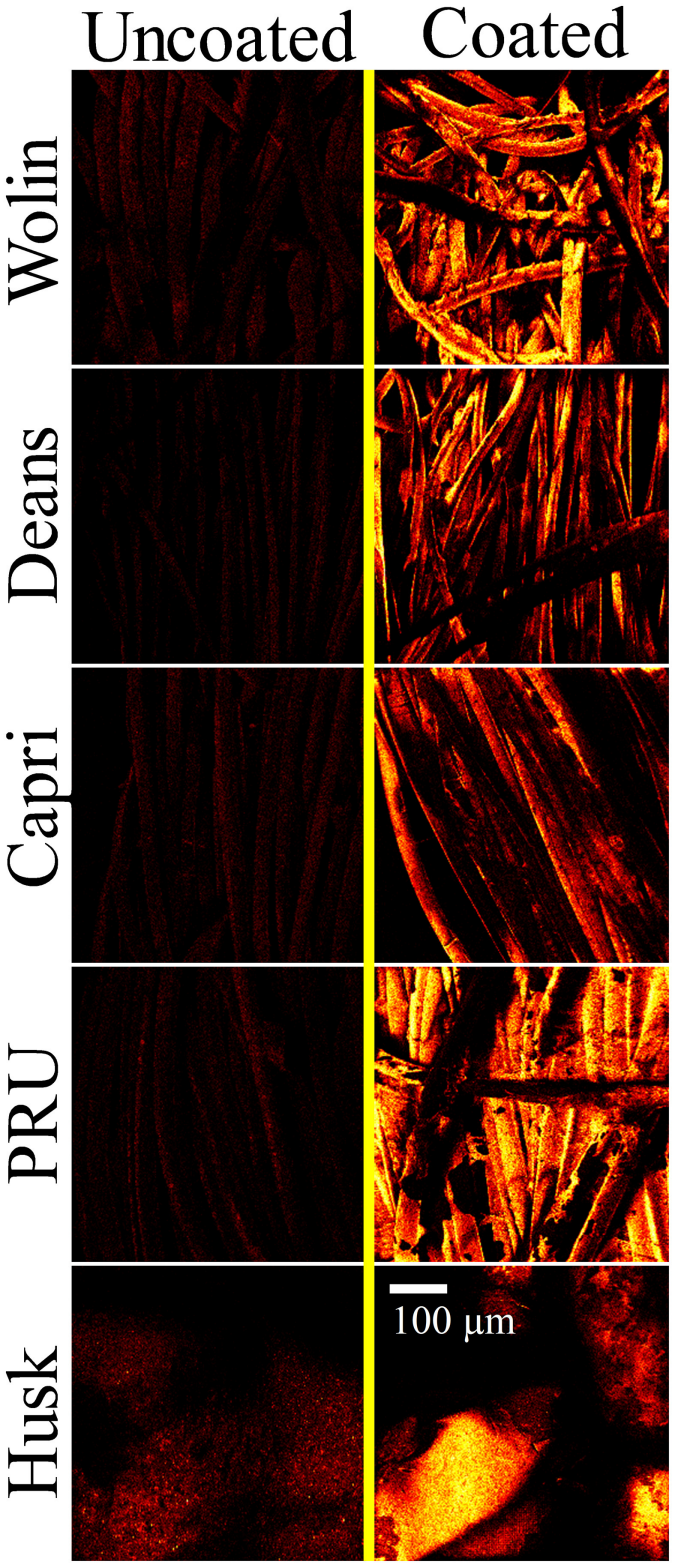
ToF-SIMS images of C_2_H_3_${\text{O}}_{2}^{-}$ ions representative of the SbQ-PVA coating acquired for the uncoated (left) and coated (right) Vescom materials with SbQ-PVA/ZnTMPyP^4+^.

Scanning electron microscopy images acquired at various magnifications (in Figure 4 and Supplementary Figure 1) confirm the presence of the SbQ-PVA layer on each of the materials. In particular, the contrast of the SEM images collected at low magnification provide insight into the successful application of the coating: the sputtered SbQ-PVA/PS-uncoated samples show significant contrast between light and dark regions, indicative of variation in the surface topology of the material, whereas the sputtered SbQ-PVA/PS-coated samples generally exhibit less surface contrast, indicative of a smoother surface topology. The PRU and Husk materials appear to possess a more even coating of SbQ-PVA, likely attributable to the pristine fibers being more closely packed/dense. Bridges and webs from the SbQ-PVA coating were also observed in images obtained from the Wolin, Deans, and Capri materials. The NIH ImageJ software package was used to analyze SEM images of the various samples to ascertain the relative thickness of the coated layer on each material. Estimates were determined by measuring the diameters of both uncoated and coated fibers and calculating the difference, which was attributed to the polymer coating. While the resultant coating thicknesses varied two-fold across the different materials [PRU (~1.8 μm) > Wolin (~1.4 μm) > Deans (~0.91 μm) > Capri (~0.76 μm)], the thickness of the coating applied to the Husk specimen could not be determined due to the lack of identifiable individual fibers at the highest magnification employed. Compared to our earlier study employing SbQ-PVA/ZnTMPyP^4+^ that yielded a coating thickness of ~150-200 nm (30), here we see a 5-10-fold increase in coating thickness. We attribute the increase here to two factors: (i) a larger quantity of initial SbQ-PVA/ZnTMPyP^4+^ solution was used to saturate the thicker Vescom materials compared with the spunbond nylon mats previously employed, resulting in a thicker photoactive base layer; and (ii) the use of two “sealant” coats (achieved by sequential spray and dip coating) on top of the base layer as opposed to a single “sealant” coat (dip only) performed in our previous study. Additional ToF-SIMS imaging was used to further demonstrate the presence of the SbQ-PVA coating (Figure 5). Comparison of pre- and post-coated materials shows a dramatic increase in C_2_H_3_${\text{O}}_{2}^{-}$ ions, confirming the uniform presence of SbQ-PVA on each sample. While previous studies (30, 39) have utilized electron dispersive x-ray spectroscopy (EDS) and ToF-SIMS to confirm the presence of the ZnTMPyP^4+^ photosensitizer, we were unable to do so here for two reasons: (i) the concentration of Zn^+^ ions, coupled with overlapping peaks at similar m/z ratios, was too low to be discerned (see section Characterization); and (ii) observation of the ZnTMPyP^4+^ Cl^−^ counterion was inconclusive to confirm the presence of the PS as each material exhibited a high loading of Cl^−^ prior to coating (data not shown).

#### Porphyrin Loading

In addition, ICP-OES was used to determine the concentration of Zn in the coated materials, which directly correlates with the amount of ZnTMPyP^4+^ photosensitizer, and thus the overall PS loading for each sample. Pristine samples were also examined to account for any background trace Zn intrinsically present within the uncoated materials. The concentration of the ZnTMPyP^4+^ PS varied ~nine-fold across all materials as follows: Husk (0.78 nmol/mg) > Wolin (0.62 nmol/mg) > PRU (0.20 nmol/mg) > Deans (0.13 nmol/mg) > Capri (0.09 nmol/mg).

### Antimicrobial Behavior

#### Employing ZnTMPyP^4+^/SbQ-PVA

**Figure 6:**
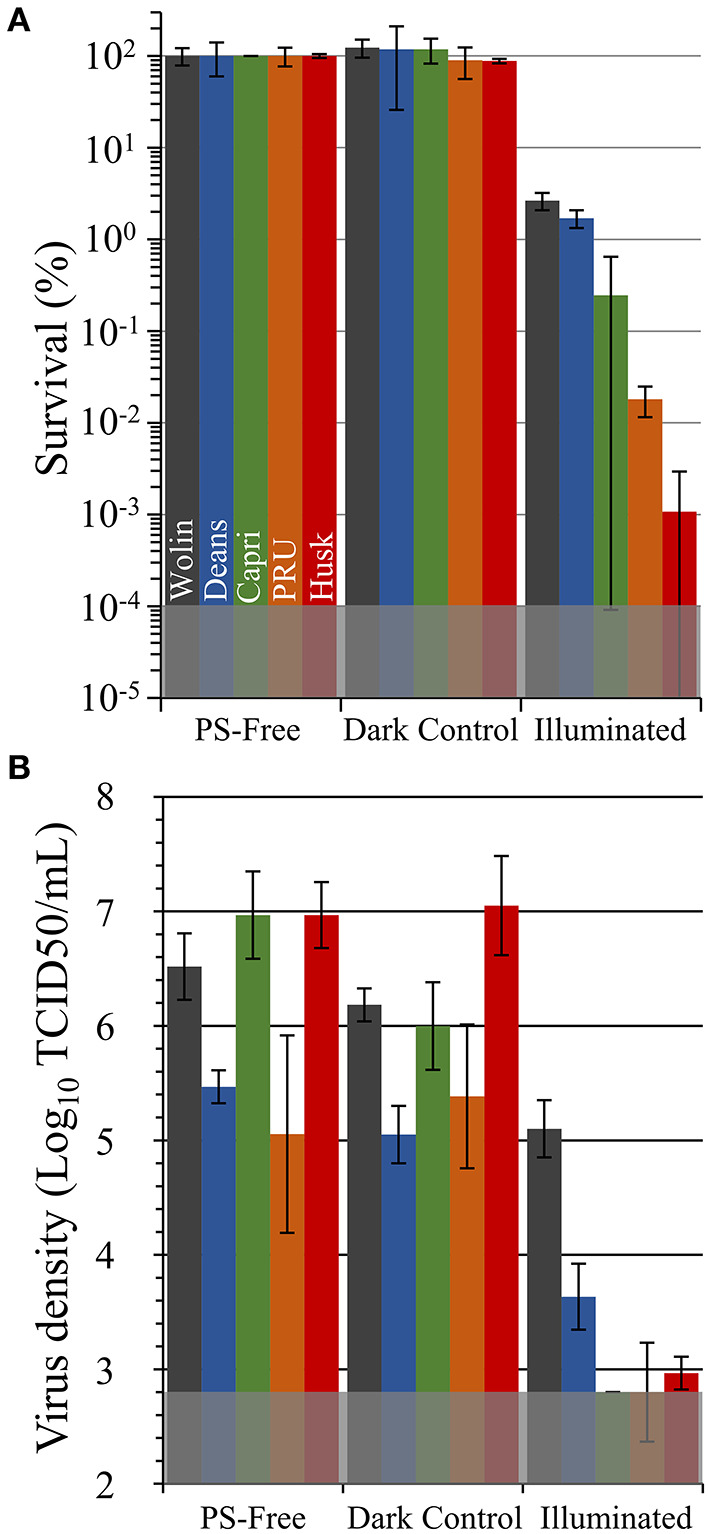
Antimicrobial photodynamic inactivation efficacy of Vescom materials coated with SbQ-PVA/ZnTMPyP^4+^ for **(A)** methicillin-susceptible *S. aureus* ATCC-29213 (MSSA) and **(B)** human coronavirus 229E (HCoV-229E). Assays were performed under fixed illumination conditions (60 min, 400-700 nm, 65 ± 5 mW/cm^2^). The gray shaded regions represent the minimum detection limit for each study. Error bars correspond to the standard deviation (*n* = 3).

Unless otherwise noted, all *in vitro* aPDI assays were performed under fixed illumination conditions (60 min, 400-700 nm, 65 ± 5 mW/cm^2^). These illumination conditions were chosen on the basis of our prior works, and we note that we have previously reported that illumination alone (e.g., light-only control) at the aforementioned intensity and duration is not sufficient to inactivate pathogens without the presence of a PS (30, 43, 44); this was again confirmed here as light-only controls did not show any statistically significant pathogen inactivation (Supplementary Figure 2). As displayed in Figure 6A, neither the SbQ-PVA-only coated materials (PS-free) nor the non-illuminated SbQ-PVA/ZnTMPyP^4+^ coated samples (dark controls) exhibited any statistically significant antibacterial activity against methicillin-susceptible *S. aureus* ATCC-29213, thereby confirming the requirement for both light and addition of PS for photodynamic inactivation of this pathogen (in an oxygen-containing environment). Upon illumination, however, a significant photodynamic effect was observed for all SbQ-PVA/ZnTMPyP^4+^ coated samples, ranging from 97 to 99.999% CFU/ml reduction, as follows: Husk (99.999%, 5 log units, *P* < 0.001) > PRU (99.98%, ~3.9 log units, *P* = 0.002) > Capri (99.75%, ~2.9 log units, *P* < 0.001) > Deans (98.3%, ~1.9 log units, *P* = 0.009) ≈ Wolin (97.3%, ~1.8 log units, *P* = 0.002). These results confirm our expectations that the SbQ-PVA/ZnTMPyP^4+^ spray coating can mediate the antibacterial photodynamic inactivation of MSSA upon illumination regardless of the base material used.

Antiviral photodynamic inactivation studies employing SbQ-PVA/ZnTMPyP^4+^-coated samples against HCov-229E are included in Figure 6B. Due to its infectivity and facile transmission, the SARS-CoV-2 virus requires at least biosafety level 3 (BSL-3) containment, which was not available for this study. For this reason, we chose to examine the HCoV-229E virus as a less pathogenic surrogate that has been documented (3, 45, 46) to possess similar environmental stability as the more pathogenic coronaviruses (SARS-CoV, MERS-CoV, and SARS-CoV-2). Similar to the antibacterial studies described above, neither the SbQ-PVA-coated materials (PS-free) nor the non-illuminated SbQ-PVA/ZnTMPyP^4+^-coated samples (dark controls) exhibited any statistically significant antiviral inactivation. In marked contrast, a substantial reduction in virus infectivity after exposure to light was observed for all spray-coated samples (these inactivation values are relative to the dark controls): Husk (99.991%, ~4 log units, *P* < 0.001) > Capri (99.995%, ~4.1 log units, *P* = 0.001) > PRU (99.8%, ~2.5 log units, *P* = 0.03) > Deans (98.3.1%, ~1.8 log units, *P* = 0.007) ≈ Wolin (96.3%, ~1.4 log units, *P* = 0.02). With the exception of the Capri and Deans specimens that switched their order, the level of antiviral activity of the coated samples mirrors that observed above in the antibacterial study.

#### Employing Other PS/SbQ-PVA Coatings

**Figure 7:**
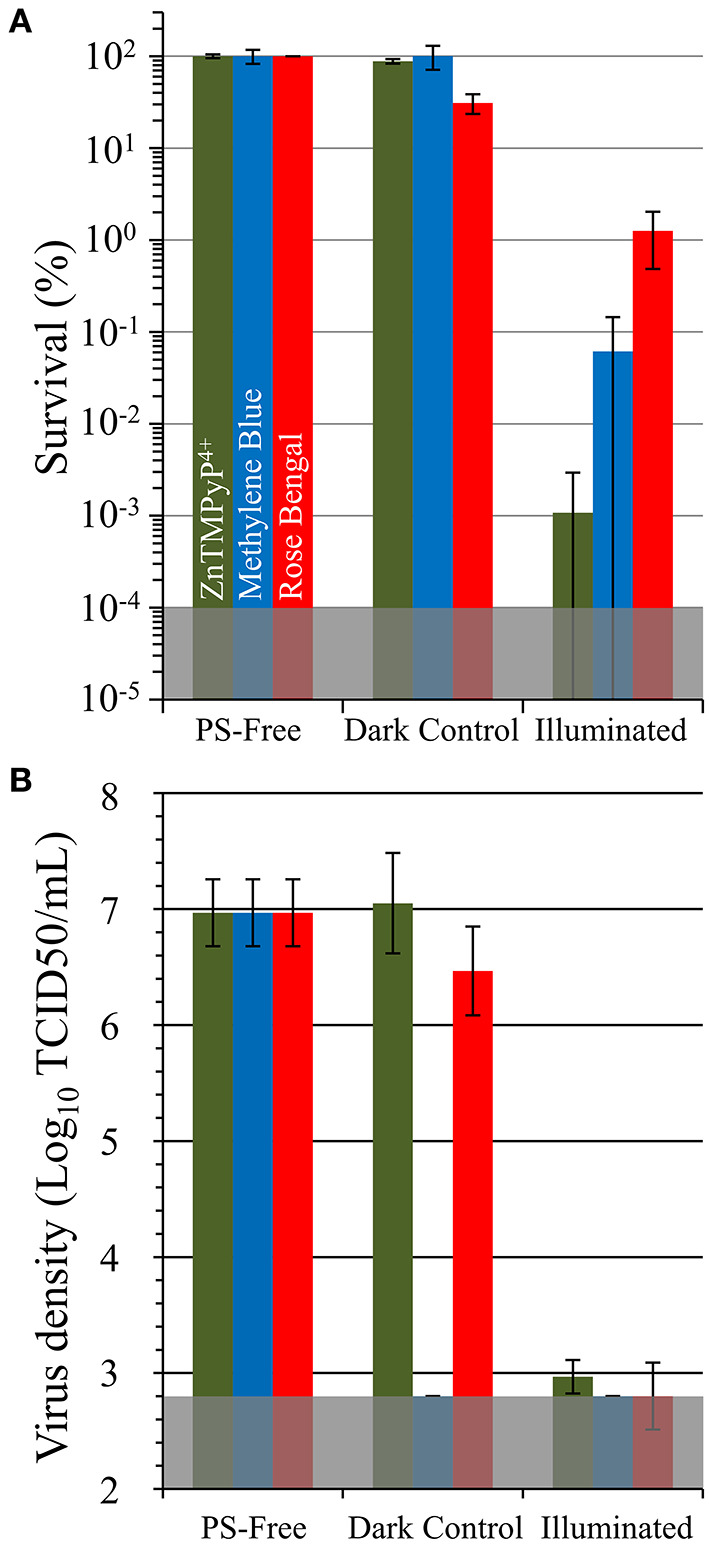
Antimicrobial photodynamic inactivation efficacy of Vescom materials coated with SbQ-PVA/MB and SbQ-PVA/RB for **(A)** methicillin-susceptible *S. aureus* ATCC-29213 (MSSA) and **(B)** human coronavirus 229E (HCoV-229E). Experimental conditions were the same as those listed in Figure 6. The results for SbQ-PVA/ZnTMPyP^4+^ are included for ease of comparison.

To examine the versatility of SbQ-PVA for accommodating a wide range of photosensitizers, we formulated two additional spray coatings for the Husk material, SbQ-PVA/methylene blue (MB) and SbQ-PVA/Rose Bengal (RB), at the identical 1 wt% PS loading level employed for SbQ-PVA/ZnTMPyP^4+^ and evaluated their aPDI efficacies against methicillin-susceptible *S. aureus* (MSSA) and HCoV-229E (Figure 7). The Husk specimen was selected as the base material as it was highly effective against both pathogens in the studies above (section Employing ZnTMPyP^4+^/SbQ-PVA). Against MSSA, an inactivation of 99.94% (3.4 log units, *P* < 0.001) was gratifyingly observed for SbQ-PVA/MB, but a lower efficacy of 98.75% (1.9 log units, *P* < 0.001) was ascertained for SbQ-PVA/RB (Figure 7A). This was not unexpected, however, as the anionic RB photosensitizer is likely to have a comparatively poorer aPDI efficacy due to electrostatic repulsion with the negatively-charged cell wall of the bacterium when compared to the cationic methylene blue PS (47–53). The comparatively lower efficacy of the MB and RB coatings vs. that employing ZnTMPyP^4+^ is consistent with previous solution studies employing these photosensitizers (42, 54). In contrast to the differential antibacterial results between these three photosensitizer coatings, both SbQ-PVA/MB (99.992%, ~4.1 log units, *P* < 0.001) and SbQ-PVA/RB (99.979%, ~3.8 log units, *P* < 0.001) were able to promote photodynamic inactivation of HCoV-229E (Figure 7B) as effectively as SbQ-PVA/ZnTMPyP^4+^.

**Figure 8:**
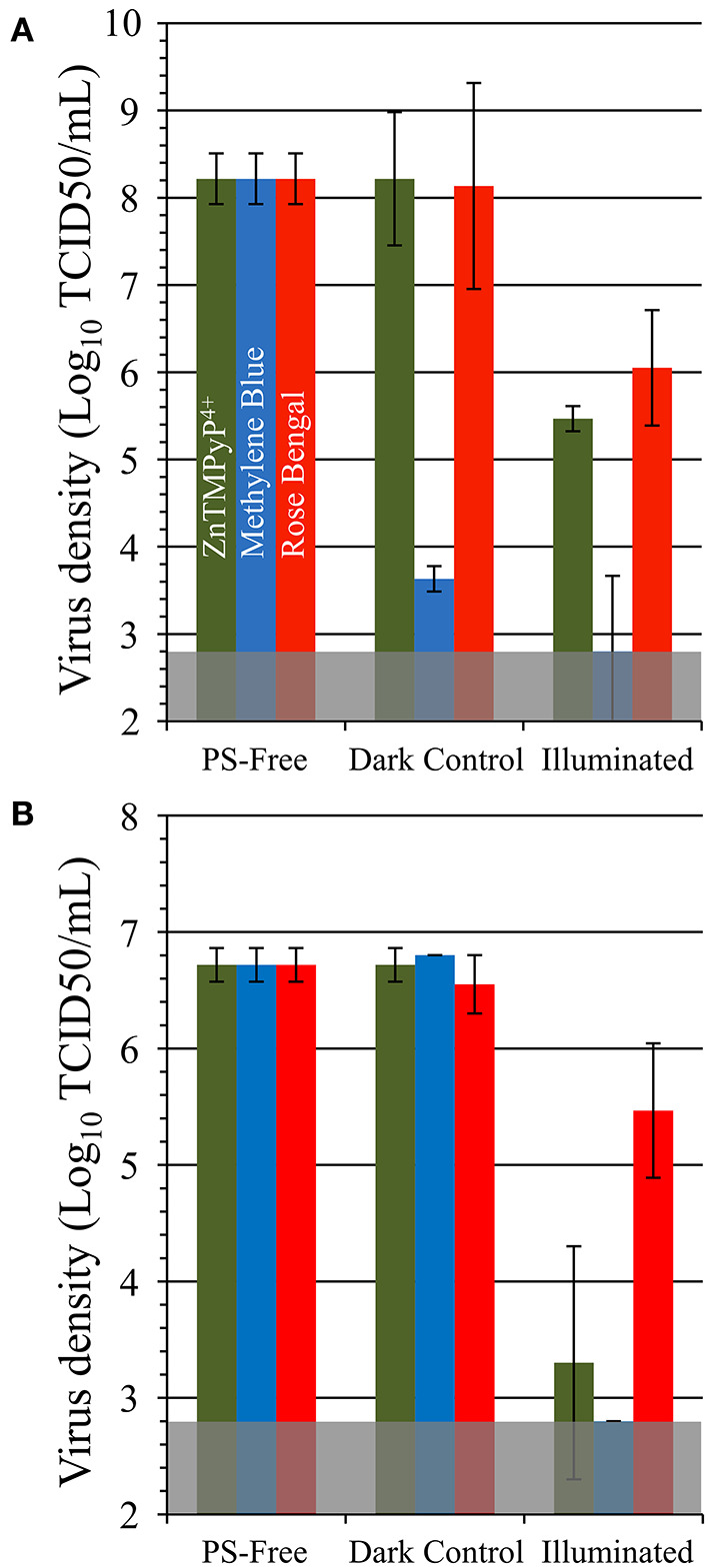
Antiviral photodynamic inactivation efficacy of Vescom materials coated with SbQ-PVA/ZnTMPyP^4+^, SbQ-PVA/MB, and SbQ-PVA/RB for **(A)** feline calicivirus (FCV) and **(B)** adenovirus. Experimental conditions were the same as those listed in Figure 6.

To determine the antiviral efficacy of the coatings against non-enveloped viruses, materials were likewise tested against feline calicivirus (FCV) and human adenovirus (Figure 8). SbQ-PVA/MB was was found to inactivate FCV by 99.998% (~5.4 log units, *P* = 0.001) relative to PS-free controls. Similar to when tested against HCoV-229E, SbQ-PVA/ZnTMPyP^4+^ and SbQ-PVA/RB had lower levels of FCV inactivation (99.8%, ~2.8 log units, *P* = 0.001; and 99.0%, 2 log units, *P* = 0.014, respectively). A similar trend in inactivation by these materials was apparent when examined against human adenovirus-5, with the SbQ-PVA/MB coating demonstrating the highest efficacy (>99.98%, >3.9 log units, *P* < 0.001), followed by SbQ-PVA/ZnTMPyP^4+^ (99.8%, ~3.4 log units, *P* = 0.014) and finally SbQ-PVA/RB (91.8%, ~1 log units, *P* = 0.04). The overall antiviral efficacy of the coated materials was lower for non-enveloped viruses compared to those that are enveloped. This is not unexpected as enveloped viruses have been shown to be more susceptible to ROS damage than non-enveloped viruses. Specifically, the lipids present on enveloped viruses are thought to act as major binding sites for PS, leading to an increase in oxidative damage and their higher susceptibility (55, 56).

#### Photobleaching and Repeatability Studies

To examine the longevity of the SbQ-PVA/PS coatings with respect to photobleaching, aging experiments were performed where the coatings described in section

**Figure 9:**
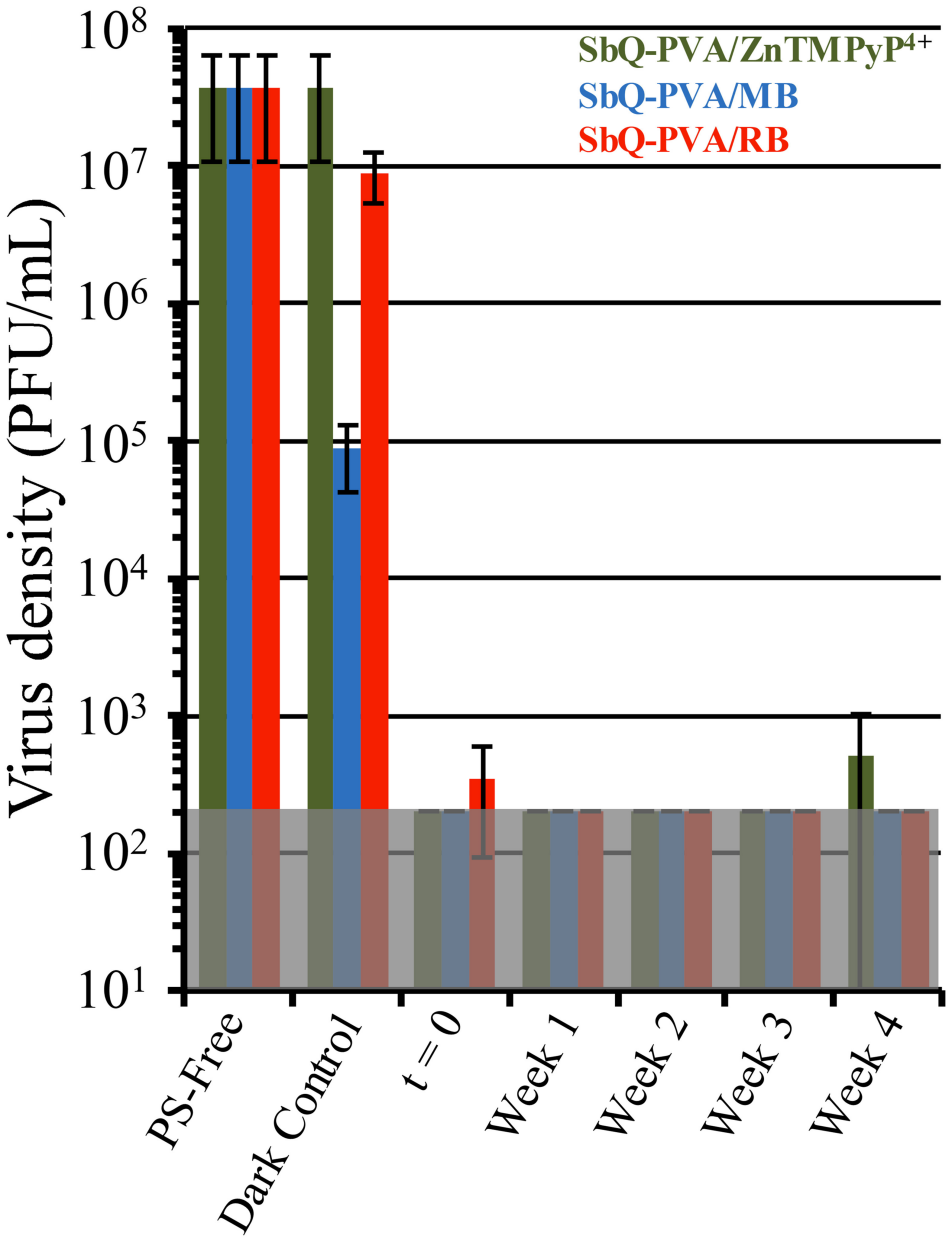
Antiviral photodynamic inactivation studies of Vescom materials coated with SbQ-PVA/ZnTMPyP^4+^ (green), SbQ-PVA/MB (blue) and SbQ-PVA/RB (red) against human coronavirus 229E (HCoV-229E). Samples were exposed to ambient room light conditions as indicated in the figure, followed by evaluation for antiviral efficacy. Results shown are for samples that had been repeatedly subjected to the aPDI assay a total of three times. Illumination conditions were the same as those listed in Figure 6.

Employing other PS/SbQ-PVA Coatings were exposed to ambient laboratory room light for 1-4 weeks, and then examined for antiviral photodynamic inactivation against HCoV-229E using our established aPDI protocol. All three spray coatings (SbQ-PVA/ZnTMPyP^4+^, SbQ-PVA/MB, and SbQ-PVA/RB), were found to inactivate HCoV-229E to the MDL regardless of their photobleaching age (data not shown). To assess repeatability, the same photobleached samples were first washed to remove traces of the initial assay, and the aPDI study against HCoV-229E was repeated a second time. Once more, all three coatings consistently exhibited inactivation to the MDL of 99.998% for these studies (data not shown). Repetition of the washing procedure followed by a third aPDI study against HCoV-229E once more yielded inactivation to the MDL (Figure 9). The results shown here confirm the longevity of the SbQ-PVA/PS coatings with respect to ambient room light photobleaching, and suggest that such coatings would remain effective over multiple pathogen exposures.

## Discussion

Both the COVID-19 pandemic and the chronic problem of HAIs illustrate that the transmission of viruses and drug-resistant bacteria from contaminated surfaces to new hosts constitutes a major threat to global healthcare, especially for elderly, injured and immune-compromised patients. Accordingly, effective preventative measures that include both surface disinfection methods and increased production of PPE must be identified and put into place. To this latter point, however, the pandemic has repeatedly highlighted critical gaps in existing healthcare and manufacturing infrastructures, especially with regard to PPE availability. With supply chains impacted by a combination of worker illness, as well as shortfalls in common raw material sources (e.g., polypropylene) due to dramatically heightened demand, medical personnel and the general population have faced critical shortages of PPE, including N95 face masks, during the COVID-19 pandemic. In fact, they have resorted to reusing disposable PPE despite the apparently underestimated threat posed by the survival of the virus on surgical masks (3). Thus, there is a critical need for re-usable and self-disinfecting PPE (SD-PPE) that is capable of affording patients and medical personnel with broad protection (against bacteria, viruses, and fungi) for prolonged periods of time, and which are cost effective and facile to manufacture. Accordingly, the results obtained both previously (30) and now here with SbQ-PVA/PS demonstrate the potential for photodynamic coatings to be more universally deployed for infection control. Spray-coating procedures (e.g., UV inks) can be easily integrated into PPE manufacturing lines; polymers such as SbQ-PVA are relatively eco-friendly (using water as the main solvent); and PSs such as MB (commercialized as Provayblue™) with extensive FDA safety information can be selected, thereby minimizing regulatory issues.

As PSs are often the highest cost component in a photodynamic coating, the efficiency of PS use is a significant consideration: PSs buried deep within a polymer beyond the diffusion limit of the ROS, e.g., <250 nm for ^1^O_2_ (57– 59), will contribute to coating cost, but not to antimicrobial efficacy. We have previously established that photodynamic materials employing cellulose-based scaffolds (cellulose nanocrystals, nanofibrillated cellulose and macrofibers/paper) generally contained PS loading levels in the range of 10-400 nmol/mg material (34, 38, 43, 44, 60–62), traditionally-dyed fabrics were in the range of 10-80 nmol PS/mg material (54, 60, 63, 64), and electrospun non-woven materials employing polyacrylonitrile and nylon scaffolds were loaded in the range of 3.4-35 nmol PS/mg material (35, 40). The majority of these modified materials had the photosensitizer incorporated during fabrication, with the PS distributed throughout the material. Here, the overall PS loading *via* spray coating was found to be 0.09-0.78 nmol/mg material, significantly lower, by as much as 10,000x than observed in the aforementioned studies. We therefore surmise that spray coating as a surface-only application is an efficient method for producing photodynamic materials, akin to core-sheath fibers that utilize the outer sheath for conferring specific properties without the need to incorporate functional agents throughout the core (65).

Despite the lower photosensitizer loadings compared to previous materials, the results of the *in vitro* aPDI assays demonstrated that SbQ-PVA/PS coatings are effective at conferring antimicrobial activity to commercially available materials, with inactivation of *S. aureus*, HCoV-229E, FCV, and adenovirus by ~2-5 log units depending on the base material employed. The decrease in antiviral efficacy of materials against non-enveloped viruses (FCV and adenovirus) relative to enveloped viruses (HCoV-229E) can be attributed to the higher tolerance of non-enveloped viruses to ROS (55, 56). While some differences in efficacy between the materials can be explained in part by PS loading (e.g., the high inactivation of Husk against both pathogens correlates well to its high PS loading), this alone fails to explain why the Wolin (second highest PS loading) material consistently shows the lowest antimicrobial activity, or why the Capri specimen has relatively good antiviral efficacy despite possessing the lowest amount of PS. Such differences in efficacy likely stem from the different surface characteristics of the Vescom materials, such as hydrophobicity, porosity and thickness. Thicker and more fibrous materials (Wolin and Deans) were generally less effective at mediating both antibacterial and antiviral photodynamic inactivation, most likely due to the inability of the SbQ-PVA/PS coating to penetrate into the interior of the fiber mats where pathogens could reside beyond the diffusion distance of ^1^O_2_ produced by the surface coating. Conversely, SEM images showed that the coated Husk and PRU materials possess lower porosity, and likely minimize the penetration of the pathogens into the PS-free interior of the fiber mats. While these are the most likely factors, additional studies that more systematically control fiber/material properties are needed to obtain a better understanding of how such material characteristics affect the antimicrobial character of the SbQ-PVA/PS coatings. Although only two pathogens were tested in this work, we expect consistent results found in the current study to translate to other pathogens from the ESKAPE HAI bacterial family based on our previous findings (30, 34–36, 38, 40).

While all three spray coatings (SbQ-PVA/ZnTMPyP^4+^, SbQ-PVA/MB and SbQ-PVA/RB) exhibited comparable antiviral activity against HCoV-229E, more significant differences in their antibacterial and non-enveloped antiviral activity were noted. We interpret these differences in light of the electrostatic properties of the photosensitizers relative to those of bacterial pathogens. Specifically, the cationic photosensitizers ZnTMPyP^4+^ and MB are expected to promote antimicrobial photodynamic inactivation *via* their electrostatic attraction to the negatively charged cell membrane wall of bacteria, helping to facilitate pathogen:coating interactions that overcome the limited diffusion distance of ^1^O_2_ from a photodynamic surface (47–53). Conversely, the anionic RB photosensitizer would disfavor such an interaction owing to electrostatic repulsion. No such electrostatic-dependent mechanism between the PS and the pathogen has been identified for antiviral photodynamic inactivation, which is consistent with our results, but we recognize that the mechanisms of aPDI against viruses are comparatively less understood than for bacteria. In light of our promising results with HCoV-229E, we also investigated the long-term viability of the SbQ-PVA/PS coatings with respect to photobleaching against this pathogen. Even after 4 weeks of ambient room light exposure, all three SbQ-PVA/PS coatings were able to promote detection-limit inactivation of HCoV-229E. Those materials also remained active through a wash and assay repeat cycle. While our previous studies (34, 35, 40) have confirmed that photobleaching is decreased when a PS is embedded within a polymer matrix, it should be noted, however, that these studies were never performed for such an extended period of time, thus further emphasizing the efficacy of SbQ-PVA/PS coatings.

In conclusion, the results here further confirm that photodynamic spray coatings afford a largely unexplored route to accelerated, effective and comprehensive antimicrobial materials, particularly for their application in reusable SD-PPE. To-date, and despite their highly potent antimicrobial activity, the adoption of such photoactive materials to reduce infection transmission in hospitals and related healthcare facilities has been hampered by the lack of a scalable and cost-effective means to manufacture them, and high-production methods for producing aPDI-based materials are needed. Here, we have demonstrated that a simple bicomponent spray coating comprised of a photocrosslinkable polymer and a photosensitizer can be applied to a range of commercially available materials used in the public sector. Not only is the spray coating method facile, inexpensive and scalable through the use of off-the-shelf components, it is well-suited for expedient integration into existing manufacturing lines that already employ similar UV-curable inks. While further studies examining the launderability and durability of such coatings are needed (as are studies on a wider scope of materials, e.g., polypropylene, more commonly used in PPE), these and other studies suggest that photodynamic spray coatings may be a simple but effective tool for reducing the transmission of pathogens in healthcare settings, thereby adding to the infection-prevention toolbox available to healthcare workers and the general public.

## Data Availability Statement

The original contributions presented in the study are included in the article/Supplementary Material, further inquiries can be directed to the corresponding author/s.

## Author Contributions

CG was responsible for materials coating, characterization, antibacterial studies, and manuscript writing. BP contributed to coating formulation and manuscript editing. SK and FS designed and performed all antiviral studies. RS was responsible for the experimental design, coating formulation, and manuscript editing. RG oversaw the project scope, experimental design, and manuscript writing/editing. All authors contributed to the article and approved the submitted version.

## Conflict of Interest

The authors declare that the research was conducted in the absence of any commercial or financial relationships that could be construed as a potential conflict of interest.

## Publisher's Note

All claims expressed in this article are solely those of the authors and do not necessarily represent those of their affiliated organizations, or those of the publisher, the editors and the reviewers. Any product that may be evaluated in this article, or claim that may be made by its manufacturer, is not guaranteed or endorsed by the publisher.
