# Supplementary material for: Toward Universal Photodynamic Coatings for Infection Control
Source: Front Med (Lausanne). 2021 Jul 28;8:657837. doi: 10.3389/fmed.2021.657837 (PMC8355428; doi:10.3389/fmed.2021.657837)
Supplement: Supplementary file 1 [file Data_Sheet_1.PDF]

Supplementary Material

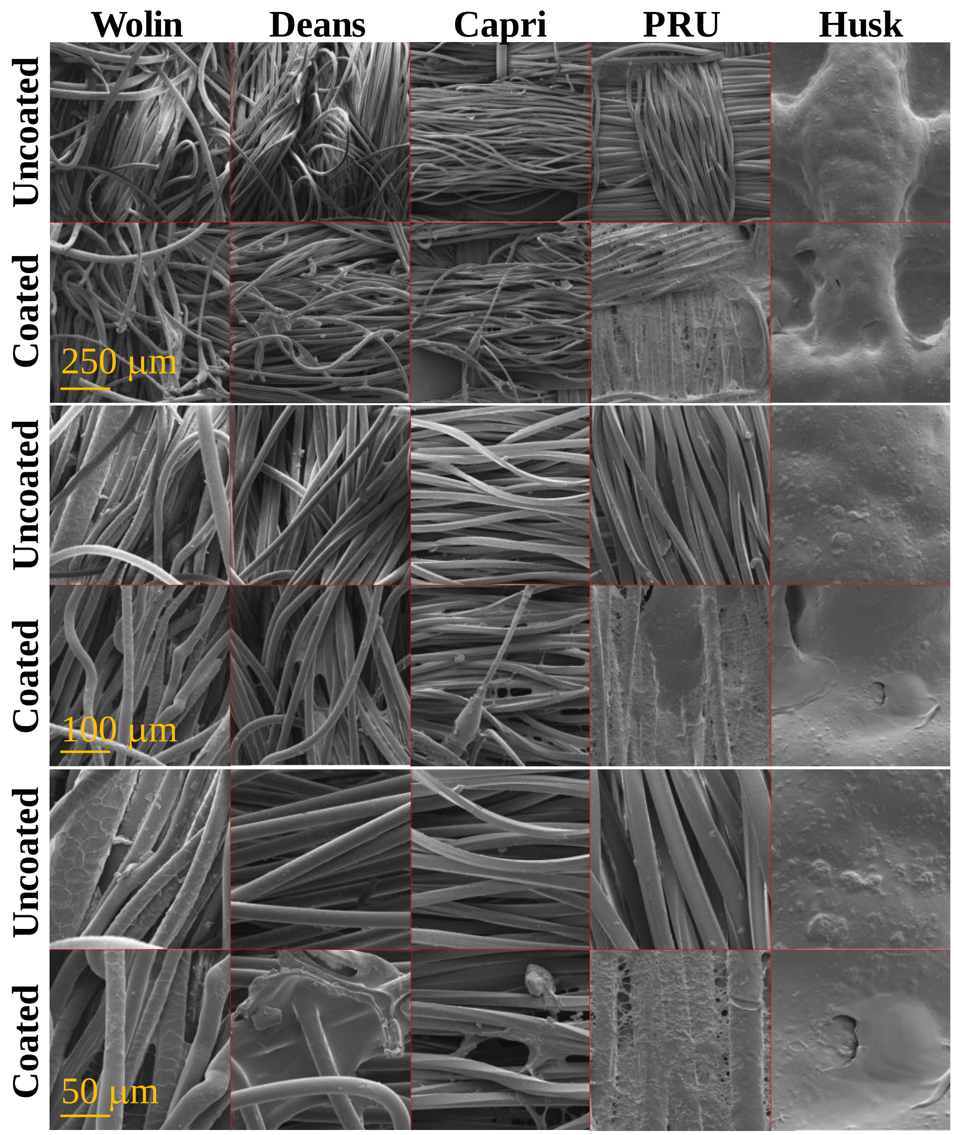

Figure S1. SEM images of uncoated and coated (SbQ-PVA/ZnTMPyP^{4+}) Vescom materials at relatively low (top), intermediate (middle) and high (bottom) magnification.

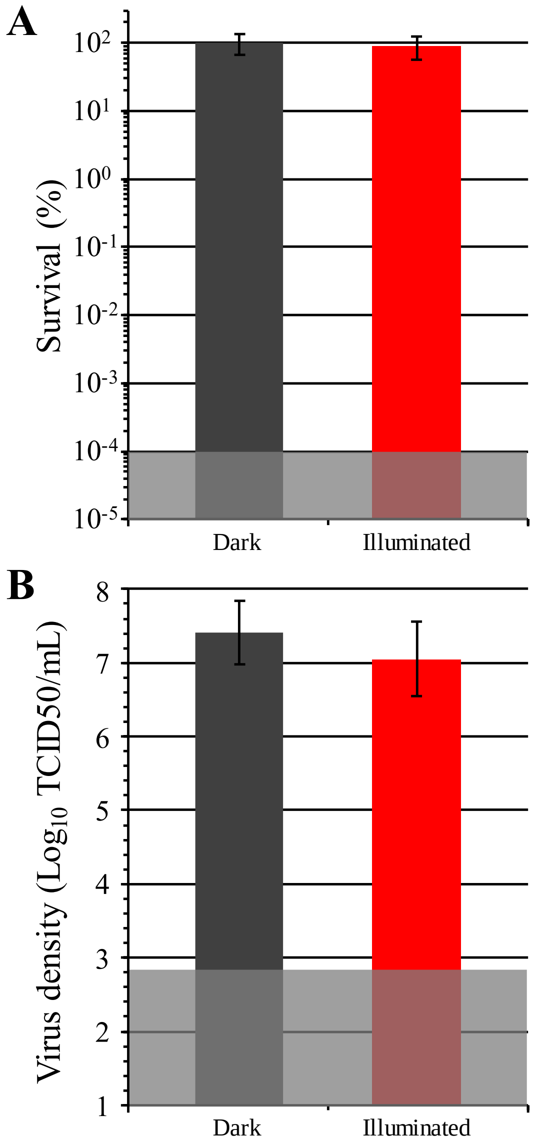

Figure S2. Effect of illumination (light-only controls) on pathogen viability for (**A**) methicillin-susceptible *S. aureus* ATCC-29213 (MSSA) and (**B**) human coronavirus 229E (HCoV-229E). Assays were performed under fixed illumination conditions (60 min, 400-700 nm, 65±5 mW/cm^2). The grey shaded regions represent the minimum detection limit for each study. Error bars correspond to the standard deviation (n = 3).
